# Structural and immunohistochemical analysis of the cellular compositions of the liver of molly fish (*Poecilia sphenops*), focusing on its immune role

**DOI:** 10.1186/s40851-022-00200-7

**Published:** 2023-01-05

**Authors:** Marwa M. Hussein, Ramy K. A. Sayed, Doaa M. Mokhtar

**Affiliations:** 1 Department of Cell and Tissues, Faculty of Veterinary Medicine, Assiut University, Assiut, 71526 Egypt; 2 Department of Anatomy and Embryology, Faculty of Veterinary Medicine, Sohag University, 82524 Sohag, Egypt; 3 Department of Histology and Anatomy, Faculty of Veterinary Medicine, Badr University in Assiut, Assiut, Egypt

**Keywords:** Hepatocytes, Kupffer cells, Macrophages, SOX9, Stem cells, Telocytes

## Abstract

The liver of fish is considered an ideal model for studying the collaboration between environmental agents and the health state of the fish, where it gives good indications about aquatic ecosystem status. Therefore, this study presented immune roles for the liver in molly fish (*Poecilia sphenops*), using immunohistochemistry and transmission electron microscopy (TEM). The hepatocytes’ sinusoidal structures of molly fish livers had taken two different forms; cord-like and tubular, while the biliary tract system showed two different types: isolated and biliary venous tract. The TEM showed that the hepatocytes possessed well-developed cytoplasmic organelles and numerous glycogen and lipid droplets of different sizes. Kupffer cells, Ito cells, aggregation of intrahepatic macrophages and melanomacrophages were also recognized. Melanomacrophages contained numerous phagosomes, many lysosomes, cytoplasmic vacuoles, and melanin pigments. Hepatocytes and Kupffer cells expressed immunoreactivity to APG5, indicating that these cells were involved in the process of autophagy. Telocytes (TCs) were also recognized in the liver of molly fish, and they shared the same morphological characteristics as those in mammals. However, TCs expressed strong immunoreactivity to APG5, TGF-β, and Nrf2, suggesting their possible role in cellular differentiation and regeneration, in addition to phagocytosis and autophagy. Both IL-1β and NF-KB showed immunoreactivity in the hepatocytes and in inflammatory cells (including intrahepatic macrophages and melanomacrophage center). Nrf2 and SOX9 showed immunoreactivity in hepatocytes, stem cells, and macrophages. The present study showed the spatial distribution of hepatic vascular-biliary tracts in molly fish. The liver of molly fish has unique functions in phagocytosis, autophagy, and cell regeneration. The expression of APG5 in hepatocytes, Kupffer cells, melanomacrophages, and telocytes supports the role of the liver in lymphocyte development and proliferation. The expression of TGF-β and NF-κB in hepatocytes, Kupffer cells, telocytes, and macrophages suggests the role of the liver in regulation of cell proliferation and immune response suppression. The expression of IL-1β and Sox9 in macrophages and melanomacrophages suggests the role of the liver in regulation of both innate and adaptive immunity, cell proliferation and apoptosis, in addition to stem cell maintenance.

## Background

*Poecilia sphenops* (Valenciennes, 1846) is a freshwater species of fish that is commonly named molly fish. They are natural inhabitants in the freshwater streams and the coastal brackish water of Mexico and Colombia. They mostly occur in swarms below the fluctuating vegetation as they feed principally on algae and other herbal resources. The mollies rank as one of the most popular feeder fish due to their high growth rate, birth size, reproduction, and brood number [1, 2].

The liver plays a crucial role in several vital processes in the living body, mainly those related to its metabolism (e.g., synthesis of plasma proteins, storage of metabolites [mainly glycogen and lipid], secretion of bile and detoxification process) that assume a dominant role in maintaining life, in addition to controlling some definite digestive processes [3, 4]. Moreover, the fish liver plays important roles in the vitellogenesis process and energy production during spawning [4, 5].

The liver is known as an immunologically complex organ that contains huge, various populations of immune cells. These cells are responsible for the production of acute phase proteins, cytokines, and chemokines [6, 7]. Moreover, the liver contains the biggest aggregations of phagocytic cells in the body, so that it can be considered as a key frontline immune tissue in the body [8].

In general, fish species present two liver forms: either associated with pancreatic tissue (hepatopancreas) or not [9, 10]. The fish hepatic structural elements, including hepatocytes, hepatic vasculature, and bile duct system, present some considerable variations in their morphological characteristics and organizations compared to mammals and between fish species too [3, 11]. These differences are not only interspecific, but they can also be recognized within the same species, varying with gender, age, water temperature, or hormonal changes correlated to the life cycle [3, 11].

Many studies have been carried out on the fish liver as it is considered an ideal model for studying the collaboration between environmental agents and the health state of the fish, where it gives good indications about aquatic ecosystem status [11, 12]. Although many liver studies have been performed on several fish species [4, 5, 9–13], there is a lack of studies concerning the cellular and stromal organization in the liver of molly fish. This study aims to identify the cellular compositions of the liver in molly fish and their role in immunity using light- and electron-microscopy and immunohistochemistry. Moreover, the study evaluates the spatial distribution of intrahepatic vascular-biliary tracts in molly fish.

## Materials and methods

The current work was performed in accordance with the Egyptian laws and University guidelines for animal care. The National Ethical Committee of the Faculty of Veterinary Medicine, Assiut University, Egypt, has approved all the procedures in the present study. The Ethical No is aun/vet/4/0015.

### Samples collection

The materials employed in this study consisted of 18 randomly obtained adult male specimens of molly fish (*Poecilia sphenops*) (10 specimens for immunohistochemistry and eight specimens for electron microscopy examination). The fish were purchased from an ornamental fish shop. The specimens were 4.20 ± 4.0 cm in standard length and 10.60 ± 1.70 gm in body weight. Healthy fish were acclimated in the laboratory for 2 weeks in aerated water tanks with a natural light/dark cycle. The swimming and feeding behavior for all studied fish were observed. Fish were randomly collected from the tanks and euthanized with an overdose of MS-222 before tissue sampling.

### Immunohistochemical analysis

Small specimens for immunohistochemical analysis were dissected and were immediately fixed in neutral buffered formalin solution (10%) for 24 h. The fixed materials were dehydrated in an ascending series of ethanol, cleared in methyl benzoate, and then embedded in paraffin wax. Tissue specimens were transverse sectioned at 3–5 μm thickness. These sections were prepared for immunohistochemical analysis using a Pierce Peroxidase Detection Kit (36,000, Thermo Fisher Scientific, UK). The sections were deparaffinized with xylene, rehydrated in graded ethanol, and washed with distilled water. The sections were heated for 15 min in sodium citrate buffer (0.01 M, pH 6.0) in a microwave to increase epitope exposure. The sections were cooled at room temperature for 30 min, washed with wash buffer (Tris-buffered saline with 0.05% Tween-20 detergent), and then were incubated for 30 min in peroxidase suppressor to quench endogenous peroxidase activity. The tissues were washed two times for 3 min with wash buffer and were blocked with universal blocker™ blocking buffer in TBS for 30 min at room temperature. The sections were incubated overnight at 4 °C with the following diluted (1:100) primary antibodies that showed reactivity in fish species: a rabbit polyclonal anti-autophagy protein 5 (APG5 or ATG5) (LS-B1843, LSBio, USA), monoclonal anti-mouse TGF-β (1:100, MA5-16949, Thermo Fischer Scientific, UK), a rabbit polyclonal anti-nuclear factor kappa B (NF-κB) (10745-1-AP, Proteintech, USA), a rabbit polyclonal anti-nuclear factor erythroid 2-related factor 2 (Nrf2) (NBP1-32822, Novus Biologicals, USA), anti-Interleukin 1 beta (IL-1β) (sc-7884, Santa Cruz Biotechnology, Heidelberg Germany), and a goat polyclonal anti-SRY-Box transcription factor 9 (Sox9) (ab82578, Abcam, Cambridge, UK,). The slides were washed two times for 3 min with wash buffer and were incubated with diluted (1:1000) goat anti-rabbit IgG (65-6140, Invitrogen, USA) or diluted (1:100) goat anti-mouse IgG (31,800, Invitrogen, USA) secondary antibodies for 30 min at room temperature. Following that, the slides were washed three times for 3 min each with wash buffer, and the tissues were incubated with the diluted (1:500) Avidin-HRP (43-4423, Invitrogen, USA) in universal blocker blocking buffer for 30 min. The slides were then washed three times for 3 min each with wash buffer. The tissues were incubated with 1X metal enhanced DAB substrate working solution (by adding stable peroxide buffer to the 10X DAB/Metal Concentrate) for 5–15 min until the desired staining was achieved. Finally, the sections were washed two times for 3 min each with wash buffer, counterstained with Harris modified Hematoxylin, and mounted with mounting media.

### Semithin sections and transmission electron microscopy (TEM)

Small specimens of the molly fish liver were fixed in a solution of 2.5% paraformaldehyde- glutaraldehyde and left overnight for fixation [14]. Then they were washed in 0.1 mol/L phosphate buffer and osmicated with 1% osmium tetroxide in 0.1 mol/L sodium-cacodylate buffer at pH 7.3. After that, the specimens were dehydrated by ethanol followed by propylene oxide and embedded in Araldite. One-µm-thick semithin sections were stained with Toluidine blue and examined under a light microscope. Ultrathin Sect. (70 nm) were obtained using an Ultrotom-VRV (LKB Bromma, Germany) and stained with lead citrate and uranyl acetate [15]. TEM images were captured with a JEOL-100CX II electron microscope.

### Digitally colored TEM images

To increase the visual contrast between several structures in the same electron micrograph, we digitally colored specific elements as hepatocytes, Ito cells, macrophages, etc., to make them more visible for the readers. All the elements were carefully hand colored using Adobe Photoshop software version 6.

## Results

### Histological analysis

**Fig. 1:**
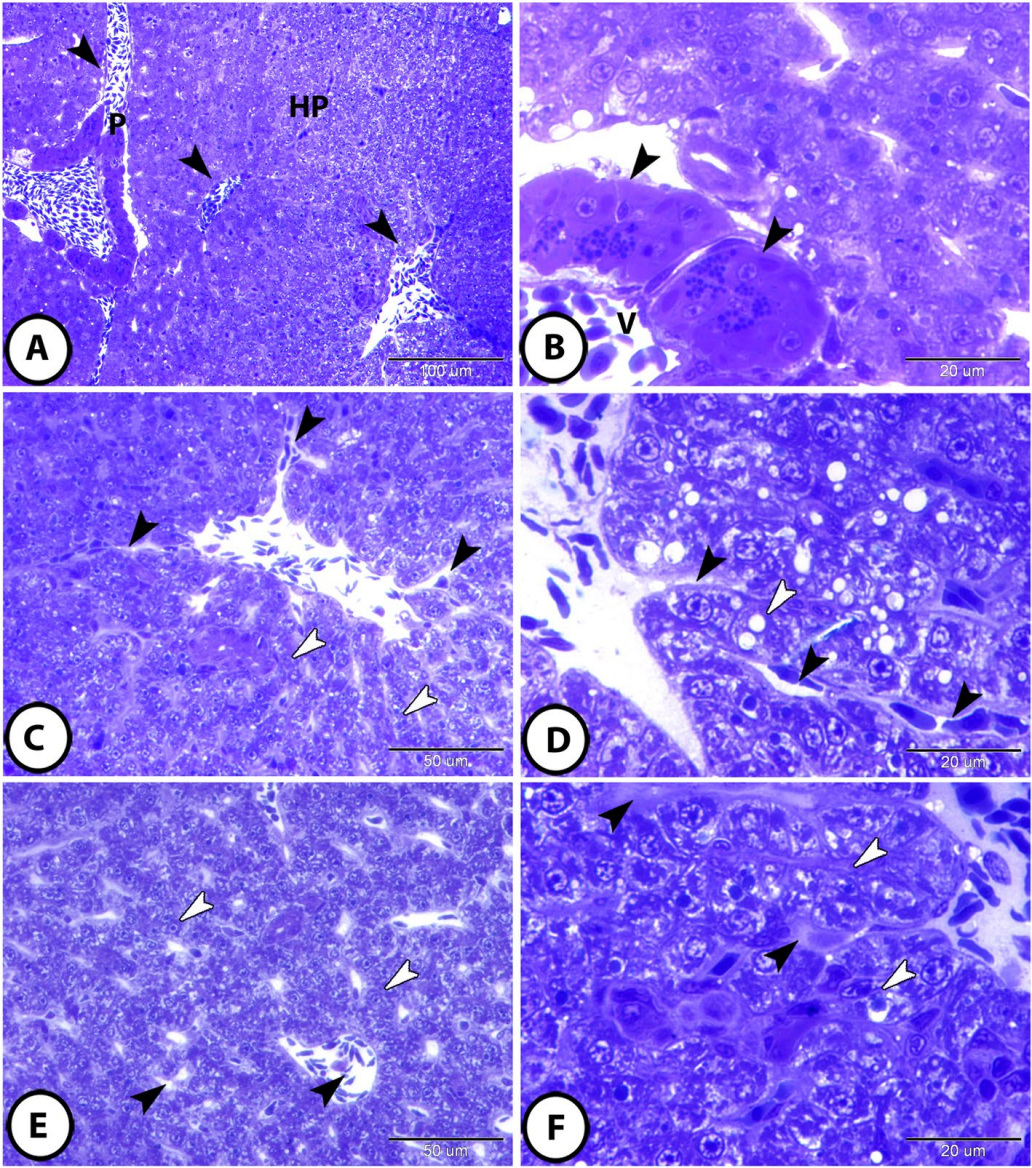
Semithin sections of the hepatic parenchyma of molly fish stained with toluidine blue. **A** The hepatic parenchyma (HP) was interrupted with islands of connective tissue that enclosed the hepatic vascular and biliary components (arrowheads) and pancreatic tissue (P). **B **The pancreatic tissue appeared as clusters of hepatopancreatic acini that were composed of groups of serous cells (arrowheads) encircling a vein (V). **C **and **D** The hepatic parenchymal arrangement showed cord-like form; most hepatocytes (white arrowheads) were arranged in simple layers and the hepatic sinusoids (black arrowheads) were relatively straight and enlarged. **E **and **F** The tubular form: many hepatocytes (white arrowheads) were arranged in double layers and the hepatic sinusoids (black arrowheads) were relatively narrow and irregular in shape

The liver of molly fish appeared as a compound organ in the form of hepatopancreas. The hepatic parenchyma was not organized into lobules and was composed of hepatocytes interrupted with connective tissue that enclosed the hepatic vascular and biliary components (Fig. 1A). The pancreatic tissue was recognized as clusters of pancreatic acini. Each hepatopancreatic acinus was composed of group of serous cells and encircled a vein that ramified up smaller branches, which then radiated into several hepatic sinusoids (Fig. 1B).

### Hepatocyte-sinusoidal structure (parenchymal arrangement)

The hepatocytes sinusoidal structures of molly fish livers showed two different forms: cord-like and tubular. In the cord-like form, many hepatocytes were arranged in simple layers and the hepatic sinusoids were relatively straight and enlarged (Fig. 1C, D). In the tubular form, most hepatocytes were arranged in double layers and the hepatic sinusoids were relatively narrow and irregular in shape (Fig. 1E, F).

### Spatial distribution of vascular and biliary components

**Fig. 2:**
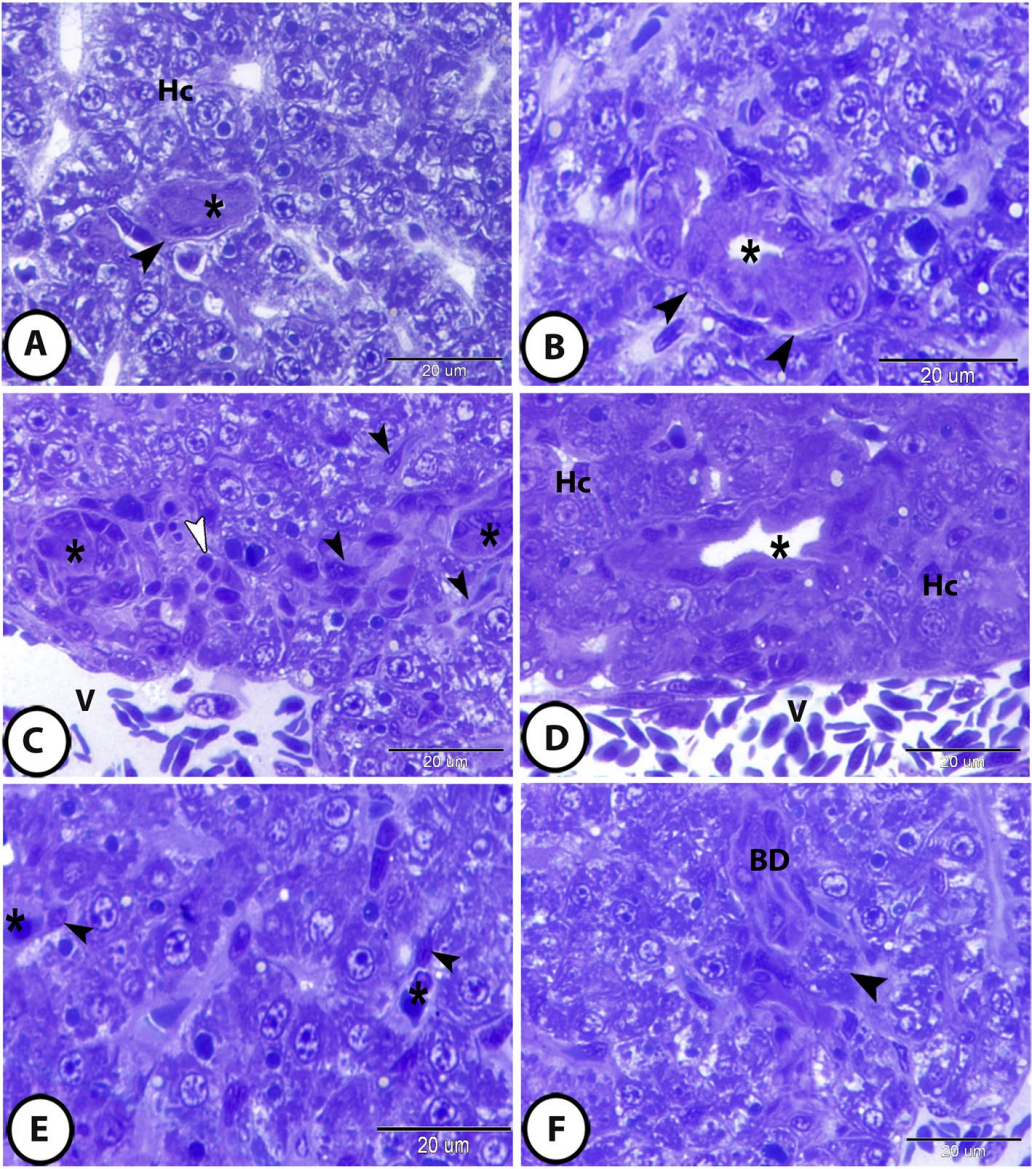
Semithin sections of the stromal and cellular constituents of molly fish liver stained with toluidine blue. **A **and **B** The biliary tract system showed isolated type of distribution, the bile ductules (asterisks) were found independently within the hepatic parenchyma and were enclosed by connective tissue sheath (arrowheads). Hepatocytes (Hc) showed vacuolated cytoplasm containing abundant glycogen and lipid droplets. **C **and **D** The biliary tract system showed the biliary venous tract (BVT) type of distribution, the bile ductules (asterisks) were supplemented with portal venules (V) and were recognized within the hepatic parenchyma. Hepatocytes (Hc) showed deeply stained cytoplasm containing deeply stained inclusions. Telocytes (black arrowheads) and some immune cells (white arrowheads) were recorded within the connective tissue sheath surrounding the bile ductule. **E** Kupffer cells (arrowheads) showed cytoplasmic processes that protruded into hepatic sinusoid (asterisks). **F** Melanomacrophage center (arrowhead) was in close topographic association to bile ductule (BD).

According to the spatial distribution of vascular and biliary components, the biliary tract system in molly fish showed two different types: isolated and biliary venous tract (BVT). In the isolated type, the bile ductules were found independently within the hepatic parenchyma and were enclosed by connective tissue sheath (Fig. 2A, B). In the BVT type, the bile ductules were supplemented with portal venules and were recognized in the hepatic lobule (Fig. 2C, D).

The intrahepatic bile ductules were lined by a single layer of high cuboidal or columnar cells that was enclosed by a thin layer of connective tissue followed by a thin layer of circularly arranged smooth muscle fibers. The duct cells showed a clearly distinct brush border (Fig. 2B, D, F).

Hepatocytes appeared as large polygonal cells with a distinct cell membrane. They contained large spherical euchromatic nuclei with distinct nucleoli. The cytoplasm of hepatocytes showed a wide variation depending on the functional activity. Some hepatocytes showed vacuolated cytoplasm containing abundant glycogen and lipid droplets (Fig. 2A); meanwhile, other hepatocytes revealed deeply stained, less vacuolated cytoplasm containing deeply stained inclusions (Fig. 2D). Telocytes (TCs) with their characteristic long telopodes were observed, in addition to the presence of melanomacrophage center and several immune cells in a close topographic association to the bile ductules (Fig. 2C, F). Kupffer cells were also recorded and showed clear cytoplasmic processes that protruded into hepatic sinusoid (Fig. 2E).

### Immunohistochemical analysis

**Fig. 3:**
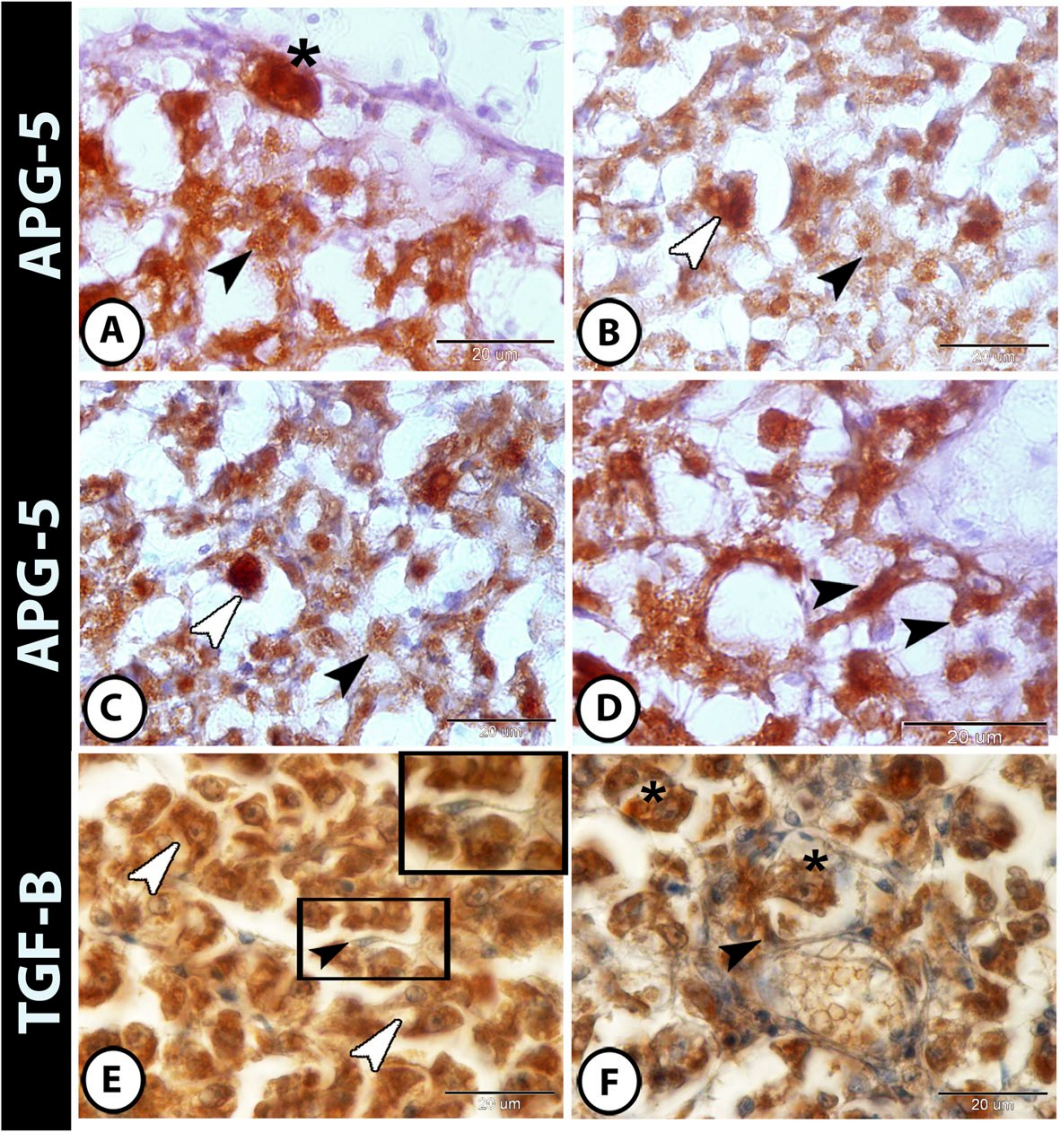
The immunohistochemistry of APG5 and TGF-β in liver of molly fish **A-C** The hepatocytes (black arrowheads) showed moderate to strong reactivity to APG5, while Kupffer cells (white arrowheads) and melanomacrophages (asterisks) showed strong reactivity to APG5. **D** Telocytes (black arrowheads) showed strong immunoreactivity to APG5 and connected to each other by their long telopodes. **E** and **F** TGF-β showed strong immunoreactivity in hepatocytes (white arrowheads), telocytes (black arrowheads), and macrophages (asterisks)

The hepatocytes showed a moderate to strong immunoreactivity to APG5 in the liver of molly fish (Fig. 3A–C), while melanomacrophages and Kupffer cells showed a strong immunoreactivity to APG5 (Fig. 3A–C). Telocytes also showed a strong immunoreactivity to APG5 and connected to each other by their long telopodes forming a network surrounding the blood vessels (Fig. 3D). TGF-β was expressed in hepatocytes, telocytes, and macrophages (Fig. 3E, F).

**Fig. 4:**
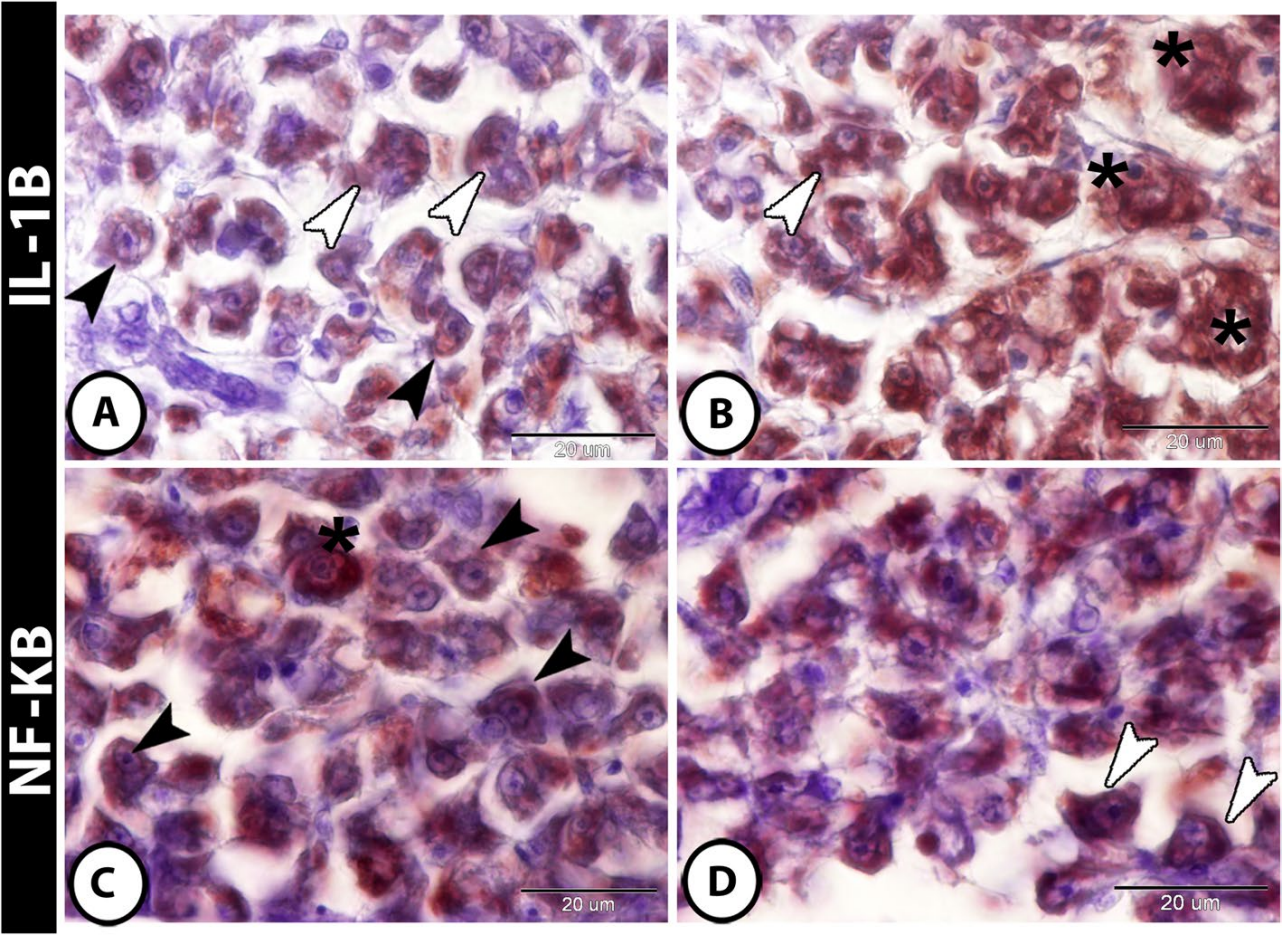
Expressions of IL-1β and NF-κB in liver of molly fish. **A **and **B** IL-1β showed strong immunoreactivity in hepatocytes (black arrowheads), macrophages (white arrowheads) and in melanomacrophage center (asterisks). **C **and **D** NF-**κ**B was strongly expressed in hepatocytes (black arrowhead), Kupffer cells (white arrowheads), and macrophages (asterisks)

**Fig. 5:**
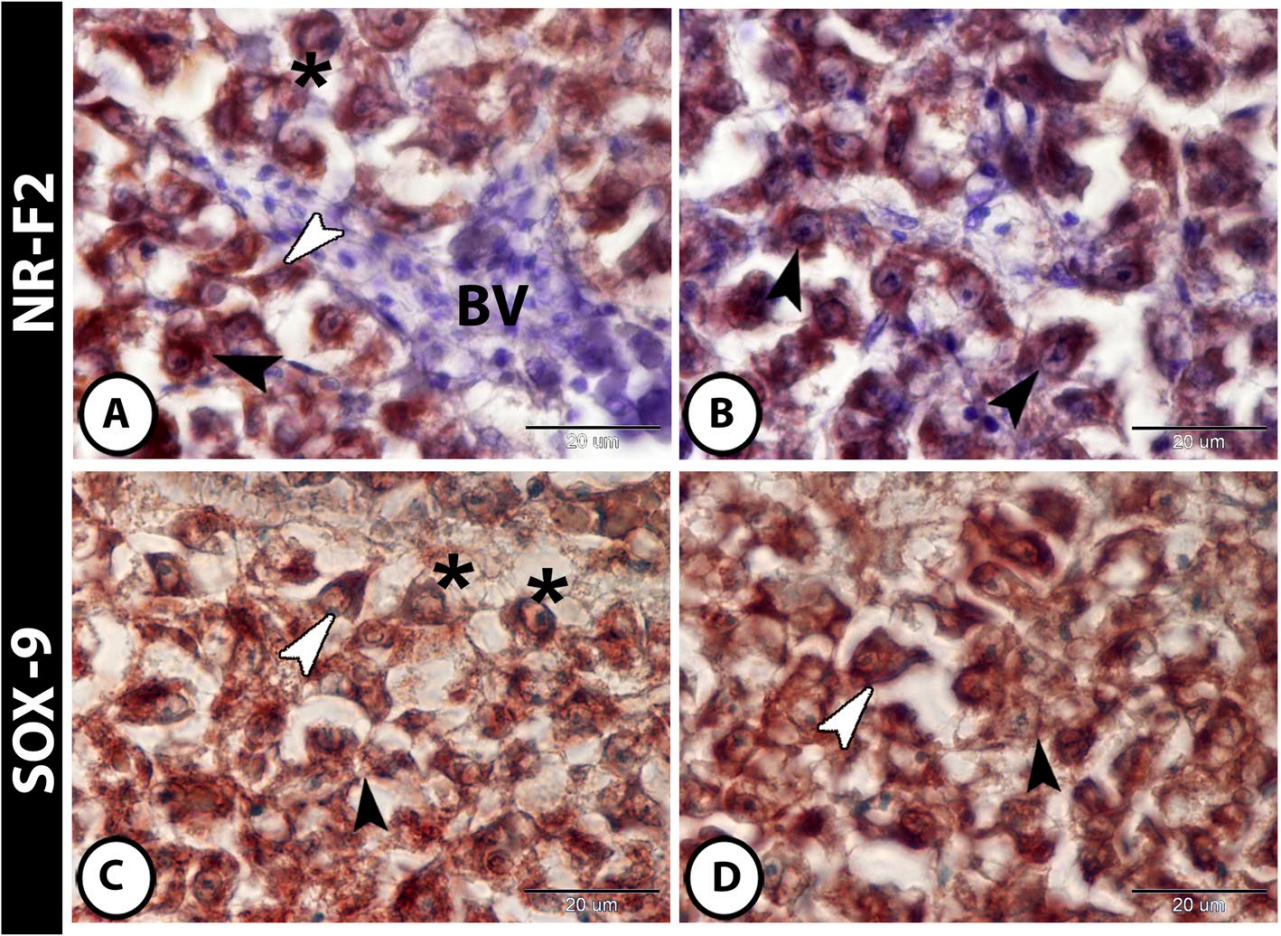
Expressions of Nrf2 and SOX9 in liver of molly fish. **A** and **B** The hepatocytes expressed Nrf2 strongly (black arrowheads). Telocytes (white arrowheads) and stem cells (asterisks) expressed NR-F2 strongly too. **C **and **D** SOX9 showed immunoreactivity in hepatocytes (black arrowheads), in stem cells (asterisks), and in macrophages (white arrowheads)

Both IL-1 β and NF-κB showed immunoreactivity in the hepatocytes and in inflammatory cells (including intrahepatic macrophages and melanomacrophage center) (Fig. 4A–D). Furthermore, hepatocytes, stem cells, and telocytes expressed NR-F2 (Fig. 5A, B). Moreover, SOX9 was expressed in hepatocytes, stem cells, and macrophages (Fig. 5C, D).

### Transmission electron microscopy (TEM)

#### Hepatocytes

**Fig. 6:**
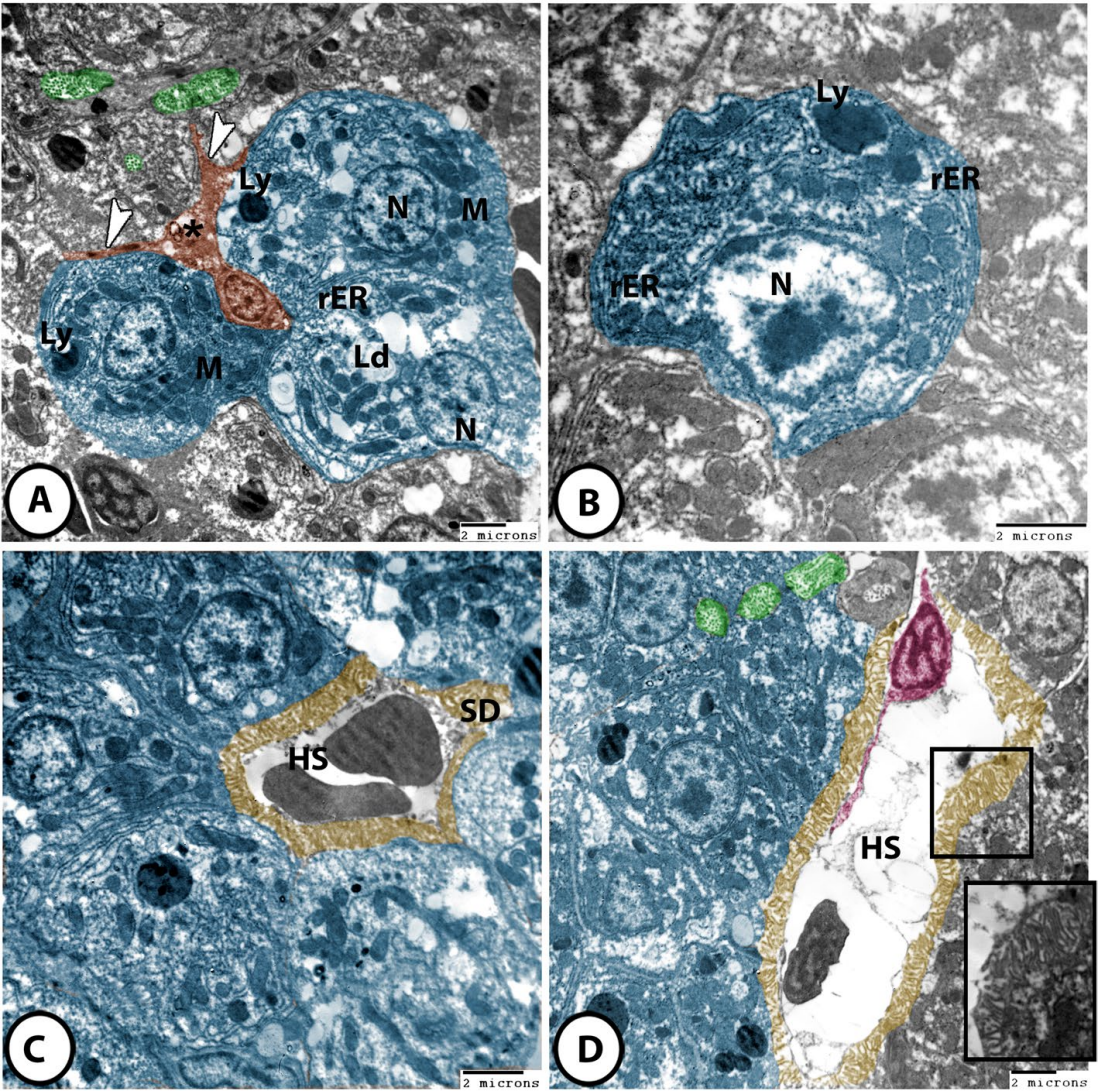
Digitally colored TEM images of the liver in molly fish. **A **and **B** The hepatocytes (blue) with large euchromatic nuclei (N), abundant mitochondria (M), many lysosomes (Ly), rough ER (rER), and lipid droplets (Ld). Ito cells (brown) intercalated between hepatocytes and showed cytoplasmic processes (arrowhead) and intracellular lipid droplets (asterisk). **C** and **D** Hepatic sinusoids (HS) surrounded with space of Disse (SD). Note Kupffer cells (pink) with their body and cytoplasmic processes. Note that the bile canaliculi (green) were made by the apposition of 2–3 hepatocytes (blue). Numerous long microvilli were projected into the lumen of bile canaliculi (yellow, boxed areas)

Hepatocytes appeared as large polygonal cells with a single large spherical euchromatic nuclei. Their cytoplasm contained abundant filamentous mitochondria, well-developed smooth endoplasmic reticulum and rough endoplasmic reticulum, many lysosomes, well-prominent Golgi complex, and numerous glycogen and lipid droplets of different sizes (Fig. 6A, B). Typical space of Disse was recognized in the hepatic cell/capillary interface. The hepatocytes’ membranes showed numerous long, prominent microvilli that projected into the space of Disse (Fig. 6C, D).

#### Intrahepatic melanomacrophages

**Fig. 7:**
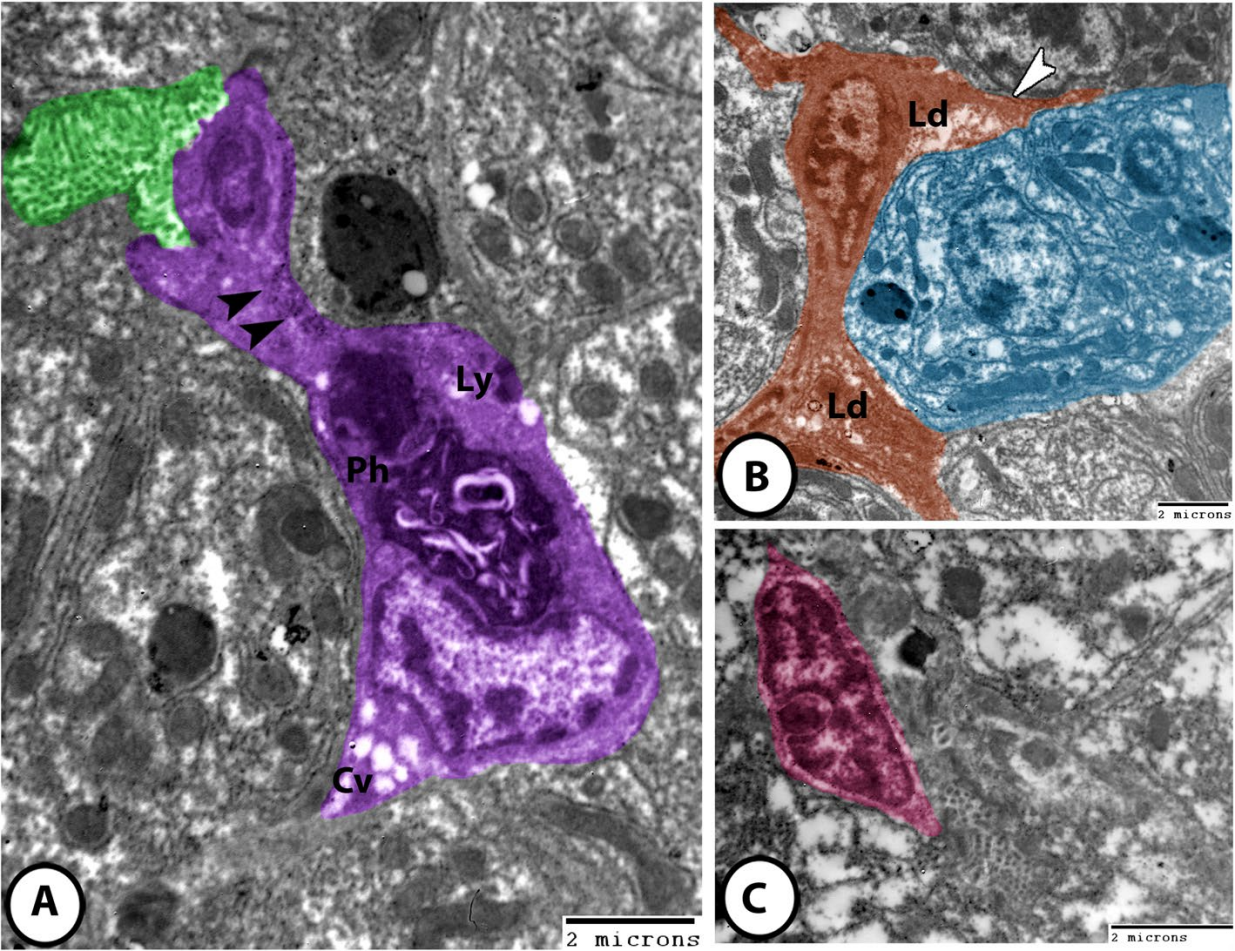
Digitally colored TEM images of the liver in molly fish. **A** Melanomacrophage center (purple) contained phagosomes (Ph), many lysosomes (Ly), cytoplasmic vacuoles (Cv), and melanin pigments (arrowheads). Note the bile canaliculi (green) located near the melanomacrophage center. **B** Ito cell (brown) intercalated between hepatocytes (blue). Note that Ito cell showed cytoplasmic processes (arrowhead) and lipid droplets (Ld). **C** Kupffer cells (pink) with their characteristic indented heterochromatic nucleus

These cells appeared as huge cells that were characterized by a large eccentric nucleus and numerous cytoplasmic processes. Their cytoplasm exhibited numerous phagocytic vacuoles, melanin-like granules, and large dense bodies containing heterogeneous material that represented phagosomes (Fig. 7A).

#### Ito cells

These cells were recognized between hepatocytes and presented numerous cytoplasmic processes. They contained large euchromatic nuclei showing clumps of heterochromatin. Their cytoplasm possessed numerous lipid droplets (Figs. 6 A, 7B).

#### Kupffer cells

They appeared as large stellate cells. They showed large heterochromatic nuclei and many cytoplasmic processes that projected into the lumen of hepatic sinusoids (Figs. 6D and 7C).

#### Bile duct system

**Fig. 8:**
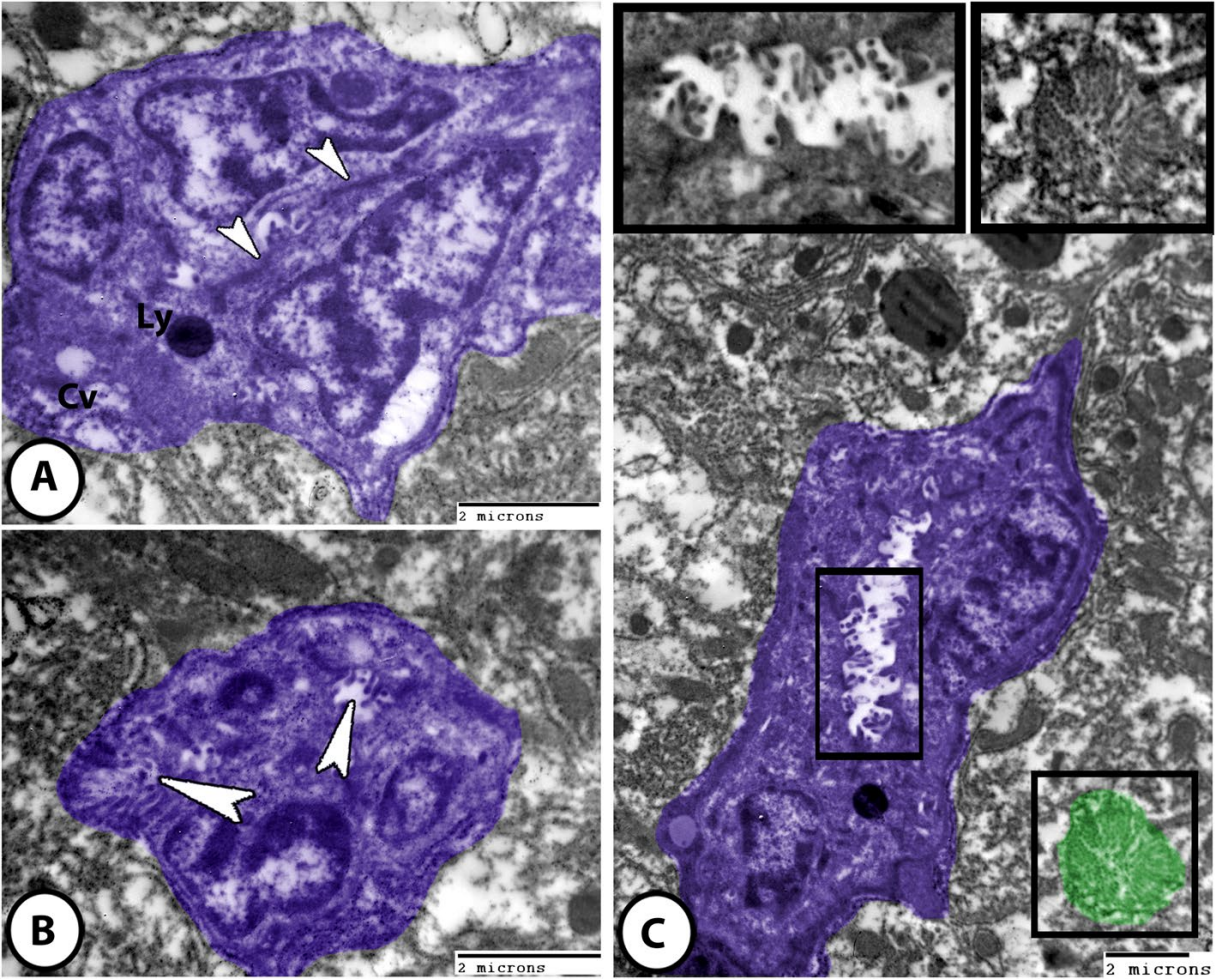
Digitally colored TEM images of the liver in molly fish. **A** The bile ductule lined with epithelial biliary cells (dark blue) that were connected to each other by desmosomes and tight junction (arrowheads). The biliary cells showed cytoplasmic vacuoles (Cv) and lysosomes (Ly). **B** The apical surface of the biliary cells showed numerous microvilli (arrowheads), which were projected into the bile ductules. **C** Bile duct (dark blue) lined by high cuboidal cells with prominent microvilli projected into the duct lumen (square boxed areas). Bile canaliculi (green) located between hepatocytes showed numerous microvilli (rectangle boxed areas)

Bile canaliculi appeared as minute channels that were formed by the membranes of adjacent hepatocytes. Numerous microvilli were projected into the lumen of bile canaliculi (Fig. 8C). The wall of the bile ductule was lined by columnar epithelial cells which were connected to each other by tight junctions and desmosomes. The cytoplasm of biliary cells showed numerous cytoplasmic vacuoles and lysosomes, while their apical surfaces showed numerous microvilli, which were projected into the bile ductules’ lumen (Fig. 8A, B). Intrahepatic bile duct was lined by high cuboidal cells with prominent microvilli that were projected into the duct lumen (Fig. 8C).

## Discussion

The current study revealed that the liver of molly fish appeared as a compound organ in the form of hepatopancreas. The hepatic parenchyma was not organized into lobules and was composed of continuous fields of hepatocytes interrupted by islands of connective tissue that enclosed the hepatic vascular and biliary components. These results agreed with those of Rocha et al. [9, 10] who stated that fish species present two liver phenotypes: either associated with pancreatic tissue (hepatopancreas) or not. Moreover, the hepatic lobules in most fish species are not well defined compared to those of higher vertebrates due to the shortening or complete absence of connective tissue septa [4].

The present study showed that the hepatocyte-sinusoidal structures of molly fish livers had taken two different forms: cord-like form and tubular form. Akiyoshi and Inoue [13] reported that the hepatocyte-sinusoidal structures of fish livers are categorized into three forms: a cord-like form, a tubular form, and a solid form. In the cord-like form, most hepatocytes are arranged in simple layers and the hepatic sinusoids are enlarged with straight capillaries. In the tubular form, most hepatocytes are arranged in double-layers and the sinusoidal capillaries are narrow and irregularly shaped. In the solid form, most hepatocytes are arranged in several layers and the hepatic sinusoids are narrow and short tortuous capillaries. Akiyoshi and Inoue [16] added that the morphology and spatial distribution of the intrahepatic vascular and biliary systems show great variation in the different fish species. These differences are not only interspecific, but they can also be recognized within the same species, varying with gender, age, water temperature, or hormonal changes correlated to the life cycle.

Bile is synthesized by hepatocytes and streams through the intrahepatic bile canaliculi, bile ductules, and bile ducts [12]. The biliary tract constructions were categorized into four types: (a) isolated type, (b) biliary-arteriolar tract (BAT) type, (c) biliary-venous tract (BVT) type, and (d) portal-tract type. The current study revealed that the biliary tract system in molly fish showed two different types: isolated type and BVT type. Akiyoshi and Inoue [16] mentioned that the BAT type was recorded in nearly all species, creating two passageways, which joined with either the isolated type or the portal-tract type. In addition, no correspondence between the bile duct morphological structures and phylogenic advancement has been reported. This indicated that fish livers have established a biliary system like that of other vertebrates.

Hepatocytes are the chief parenchymal cells in the liver, and they perform very essential roles in metabolism, detoxification, and protein synthesis [5, 17]. Hepatocytes also stimulate innate immunity against invading pathogens by synthesizing and releasing innate immunity proteins [18]. The current study revealed that the morphological characteristics of hepatocytes in the liver of molly fish are typical of those in mammalian liver. Their cytoplasm showed abundant filamentous mitochondria, well-developed smooth endoplasmic reticulum and rough endoplasmic reticulum, many lysosomes, well-prominent Golgi complex, and numerous glycogen and lipid droplets of different sizes. Similar findings were reported by many authors for many fish species, such as Teleostei, Salmonidae, grass carp or *Ctenopharyngodon idella* and Atlantic salmon [4, 9, 11].

An interesting finding in this study is the expression of APG5 in hepatocytes, Kupffer cells, melanomacrophages, and telocytes, indicating the involvement of these cells in the process of autophagy in the liver of molly fish. Autophagy is a highly synchronized biological process that is associated with stress adaptation induced by nutritional or trophic deficiencies to preserve cellular hemostasis by reprocessing the damaged organelles. APG5 is one of the essential players of the autophagy process [19], and it is critical for multiple processes including autophagic vesicle formation, lymphocyte development and proliferation, mitochondrial quality control, and apoptosis [20]. Autophagy is considered to be a protective system for the cells as it inhibits the accumulation of toxic proteins; it plays a vital role in innate and adaptive immunity, and fortification against some diseases, and aging [21]. In addition, autophagy activates apoptosis through different apoptotic mechanisms [22].

Another interesting finding in this study is the expression of TGF-β in hepatocytes, telocytes, and macrophages. Transforming growth factor-β (TGF-β) is a well-recognized, multifunctional cytokine transforming growth factor. TGF-β mediates hepatic stellate cell and fibroblast activation resulting in generation of myofibroblasts and deposition of extracellular matrix [23]. It is a pleiotropic cytokine produced by a wide variety of cells including immune cells and non-hematopoietic cells. It has significant impacts on cell proliferation, oncogenesis, and immune response suppression, in addition to suppression of intestinal inflammatory responses to bacterial antigens [24]. TGF-β plays a critical immunoregulatory role in mammals both in the innate and adaptive immune pathways [25], and it has been reported to regulate the active and inactive states of macrophages and monocytes under specific conditions [26]. Higher expression of TGF-β1 was detected in immune-associated tissues of fish, including the spleen, thymus, and head kidney [27].

One of the most striking findings in this study is the recognition of Kupffer cells, which were typical to those of mammalian liver in their morphological features, Kupffer cells have not been recognized in many teleosts. These polymorphous cells are rare and hardly recognized in fish liver. When present, they show a strong phagocytic activity [28]. Kupffer cells were recognized in spotted pimelodus [29] and in Juvenile crocodile [30]. However, they were not observed in the liver of *Kareius bicoloratus* [31] or *Salmo trutta fario* [10].

Intrahepatic macrophages, Kupffer cells, and melanomacrophage centers are all present in the liver of molly fish. They can be distinguished by their topographical location and their morphology. The intrahepatic macrophages were located between the hepatocytes and characterized by many cytoplasmic processes. Their cytoplasm characteristically displayed many small dense vesicles (lysosomes), phagocytic vacuoles, and huge dense bodies with a heterogeneous content that represented phagososomes. Kupffer cells were pleomorphic cells, situated in the hepatic sinusoids. They projected slightly to the sinusoidal lumen and established close contact with endothelial cells. They possessed irregular cell surfaces and contained lysosomes, phagosomes in the form of vacuoles, as well as a few fat droplets. The nuclei of the Kupffer cells were indented and frequently eccentrically located. Clumps of heterochromatin were distributed throughout the nuclei and formed a distinct rim along the nuclear envelope. Melanomacrophage centers (MCs) were heterogeneous in composition, and widely distributed in the liver of molly fish: between hepatocytes, around bile ducts, in the adventitia of blood vessels, and associated with pancreatic–venous complexes, like those found in other fish species [32–34]. Moreover, melanomacrophages were characterized by pseudopodia-like processes and eccentric nucleus, and their cytoplasm contained numerous melanin-like granules.

By TEM, Kupffer cells showed large vesicular nuclei and many cytoplasmic processes that projected into the lumen of hepatic sinusoids in the liver of molly fish. In addition, they expressed strong immunoreactivity to APG5, TGF-β, and NF-kB. NF-kB responds to inflammatory and immune stimuli and regulates cell proliferation, adhesion, invasion, apoptosis, and angiogenesis in multiple cell types [35]. Moreover, its signaling within epithelial cells plays fundamental roles for maintaining immune homeostasis in barrier tissues [36]. In the innate immune response, NF-κB is a critical transcription factor that mediates production of many pro-inflammatory cytokines and plays a fundamental role in various signaling pathways [37]. The findings of this study support the notion that that Kupffer cells play a vital role in autophagy and phagocytosis. These suggestions are in agreement with van Wilpe and Groenewald [30], who stated that Kupffer cells play a critical role in elimination of degenerated blood cells, degradation of hemoglobin, and removal of toxic materials.

Aggregations of intrahepatic macrophages and melanomacrophages were extensively distributed in the liver of molly fish. TEM revealed that the cytoplasm of these cells exhibited numerous phagocytic vacuoles, melanin-like granules and large, dense bodies containing heterogeneous materials that represented phagosomes. In addition, they expressed APG5, TGF-β, IL-1β, NF-κB, and SOX9. IL-1β is a critical early response proinflammatory cytokine that mediates immune regulation in both innate and adaptive immunity. It could be secreted by activated endothelial cells, tissue macrophages, blood monocytes, activated T lymphocytes, granulocytes, and other cell types [38]. It affects almost every cell type, where it plays an essential role in the initiation of systemic and local responses to infection or injury by activating macrophages, T and B lymphocytes, and natural killer cells [39, 40]. The Sox family plays essential roles in stem cell maintenance, embryonic development, and lineage commitment [41], where sox9 regulates stem and progenitor cells in adult tissues [42].

Aghaallaei et al. and Agius and Roberts [17, 43] added that these melano-macrophage aggregations resemble the morphology of Kupffer cells and show a clear phagocytic activity. Mokhtar [4] found that macrophage aggregations in the liver of grass carp are heterogeneous in composition and their cytoplasm contains iron, melanin, lipofuscin, lipid, and glycogen. Moreover, Agius and Roberts [43] suggested that these aggregates could be a site of melanin synthesis rather than storage. Recently, melano-macrophages have been reported as a histological indicator of immune function in fish and other poikilotherms [44]. They are considered one of the potential biomonitoring tools for determining the impacts of minute concentrations of pesticide contaminants [45–48], where an increase of melano-macrophage aggregates can serve as a biomarker of toxic effect [49].

Telocytes (TCs) are identified as a peculiar cell type of interstitial cells that is characterized by extremely long and thin cellular processes that are called telopodes (Tp) [50]. These cells were identified in many different tissues and organs in humans, animals, birds, and fish. It was proved that they perform a wide range of very important biological functions [51–55]. Telocytes’ distribution was first recognized in the fish liver by Mokhtar [4], who recorded them around the bile ductules and hepatic blood vessels of grass carp. In agreement with [4], the current study revealed that telocytes in fish shared the same morphological characteristics those in mammals. However, one of the most interesting observations in this study is that telocytes expressed a strong immunoreactivity to APG5, TGF-β, and Nrf2. Nrf2 has been shown to be involved in osmoregulation, antioxidation, and immunopotentiation in fish under salinity stress [56, 57]. These data suggest that telocytes can play a role in cellular differentiation and regeneration in addition to phagocytosis and autophagy.

## Conclusion

The present study showed the spatial distribution of hepatic vascular-biliary tracts in molly fish. The liver of molly fish is well organized with many types of active cellular elements. It has unique functions in phagocytosis, autophagy, and cell regeneration. The expression of APG5 in hepatocytes, Kupffer cells, melanomacrophages, and telocytes indicates the involvement of these cells in the process of autophagy and supports the possibility of a role of the liver in lymphocyte development and proliferation. The expression of TGF-β and NF-κB in hepatocytes, Kupffer cells, telocytes, and macrophages suggests the role of liver in regulation of cell proliferation, and immune response suppression. The expression of IL-1β and Sox9 in macrophages and melanomacrophages suggests a role of the liver in regulation of both innate and adaptive immunity, and in cell proliferation and apoptosis, in addition to stem cell maintenance. The expression of Nrf2 in the telocytes suggests a role of the liver in immunopotentiation and oxidative stress.

## Data Availability

Datasets analyzed during the current study are available from the corresponding author upon reasonable request.

## Abbreviations

APG5: Autophagy protein 5
TGF-β: Transforming growth factor beta
NF-κB: Nuclear factor kappa B
Nrf2: Nuclear factor erythroid 2-related factor 2
IL-1β: Interleukin 1 beta
Sox9: SRY-Box transcription factor 9
TCs: Telocytes
Tps: Telopodes

## References

[1] Alda F, Reina RG, Doadrio I, Bermingham E (2013). Phylogeny and biogeography of the *Poecilia sphenops* species complex (Actinopterygii, Poeciliidae) in Central America. Mol Phylogenetics Evol.

[2] Tembo RN (2009). The sublethal effects of low-pH exposure on the chemoreception of Poecilia sphenops. Arch Environ Contam Toxicol.

[3] Giari L, Manera M, Simoni E, Dezfuli BS (2007). Cellular alterations in different organs of european sea bass *Dicentrarchus labrax* (L.) exposed to cadmium. Chemosphere.

[4] Mokhtar DM (2018). Cellular and stromal elements organization in the liver of grass carp, *Ctenopharyngodon idella* (Cypriniformes: Cyprinidae). Micron.

[5] Yao Y, Lin J, Yang P, Chen Q, Chu X, Gao C (2012). Fine structure, enzyme histochemistry, and immunohistochemistry of liver in zebrafish. Anat Rec.

[6] Crispe IN (2009). The liver as a lymphoid organ. Annu Rev Immunol.

[7] O’Farrelly C, Crispe IN (1999). Prometheus through the looking glass: reflections on the hepatic immune system. Immunol Today.

[8] Kubes P, Jenne C (2018). Immune responses in the liver. Annu Rev Immunol.

[9] Rocha E, Monteiro RA, Pereira CA (1997). Liver of the brown trout, *Salmo trutta* (Teleostei, Salmonidae): a stereological study at light and electron microscopic levels. Anat Rec.

[10] Rocha E, Monteiro RAF, Pereira CA (1995). Microanatomical organization of hepatic stroma of the brown trout, *Salmo trutta fario* (Teleostei, Salmonidae): a qualitative and quantitative approach. J Morphol.

[11] Robertson JC, Bradley TM (1992). Liver ultrastructure of juvenile Atlantic salmon (*Salmo salar*). J Morphol.

[12] Figueiredo-Fernandes AM, Fontaínhas-Fernandes AA, Monteiro RA, Reis-Henriques MA, Rocha E (2007). Spatial relationships of the intrahepatic vascular-biliary tracts and associated pancreatic acini of Nile tilapia, *Oreochromis niloticus* (Teleostei, Cichlidae): a serial section study by light microscopy. Ann Anat.

[13] Akiyoshi H, Inoue A (2004). Comparative histological study of teleost livers in relation to phylogeny. Zool Sci.

[14] Karnovsky JM (1965). A formaldehyde glutaraldehyde fixative of high osmolality for use in electron microscopy. J Cell Biol.

[15] Reynolds ES (1963). The use of lead citrate at high pH as an electron-opaque stain in electron microscopy. J Cell Biol.

[16] Akiyoshi H, Inoue AM (2012). Comparative histological study of hepatic architecture in the three orders amphibian livers. Comp Hepatol.

[17] Aghaallaei N, Bajoghli B, Schwarz H, Schorpp M, Boehm T (2010). Characterization of mononuclear phagocytic cells in medaka fish transgenic for a cxcr3a:gfp reporter. Proc Natl Acad Sci USA.

[18] Mokhtar DM, Abdelhafez EA (2021). An overview of the structural and functional aspects of immune cells in teleosts. Histol Histopathol.

[19] Yao Z, Delorme-Axford E, Backues SK, Klionsky DJ (2015). Atg41/Icy2 regulates autophagosome formation. Autophagy.

[20] Pierdominici M, Vomero M, Barbati C, Colasanti T, Maselli A, Vacirca D (2012). Role of autophagy in immunity and autoimmunity, with a special focus on systemic lupus erythematosus. FASEB J.

[21] Abdel-Maksoud FM, Hussein MT, Attaai A (2019). Seasonal variation of the intraepithelial gland in camel epididymis with special reference to autophagosome. Microsc Microanal.

[22] Su M, Mei Y, Sinha S (2013). Role of the crosstalk between autophagy and apoptosis in cancer. J Oncol..

[23] Dooley S, Ten Dijke P (2012). TGF-β in progression of liver disease. Cell Tissue Res.

[24] Di Sabatino A, Pickard KM, Rampton D, Kruidenier L, Rovedatti L, Leakey NA (2008). Blockade of transforming growth factor β upregulates T-box transcription factor T-bet, and increases T helper cell type 1 cytokine and matrix metalloproteinase-3 production in the human gut mucosa. Gut.

[25] McGeachy MJ, Cua DJ (2007). T cells doing it for themselves: TGF-β regulation of Th1 and Th17 cells. Immunity.

[26] Ashcroft GS (1999). Bidirectional regulation of macrophage function by TGF-β. Microbes Infect.

[27] Sayed RKA, Zaccone G, Capillo G, Albano M, Mokhtar DM (2022). Structural and functional aspects of the spleen in molly fish *Poecilia sphenops* (Valenciennes, 1846): synergistic interactions of stem cells, neurons, and immune cells. Biology..

[28] Munshi JD, Dutta HM (1996). Fish morphology: Horizon of New Research.

[29] Ferri S, Sesso A (1981). Ultrastructural study of the endothelial cells in teleost liver sinusoids under normal and experimental conditions. Cell Tissue Res.

[30] van Wilpe E, Groenewald HB (2014). Kupffer cell structure in the juvenile Nile crocodile, *Crocodylus niloticus*. J Morph.

[31] Tanuma Y, Ohata M, Ito T (1982). Electron microscopic study on the sinusoidal wall of the liver in the flatfish, *Kareius bicoloratus*: demonstration of numerous desmosomes along the sinusoidal wall. Arch Histol Jpn.

[32] Rocha E, Monteiro RA, DNE Saksena (1999). Histology and cytology of fish liver: a review. Ichthyology: Recent Research Advances.

[33] Coimbra AM, Figueiredo-Fernandes A, Reis-Henriques MA (2007). Nile tilapia (*Oreochromis niloticus*), liver morphology, CYP1A activity and thyroid hormones after Endosulfan dietary exposure. Pestic Biochem Physiol.

[34] Sales CF, Silva RF, Amaral MG, Domingos FF, Ribeiro RI, Thomé RG, et al. Comparative histology in the liver and spleen of three species of freshwater teleost. Neotropi Ichthyol. 2017;15(1):e160041.

[35] Viatour P, Merville M-P, Bours V, Chariot A (2005). Phosphorylation of NF-κB and IκB proteins: implications in cancer and inflammation. Trends Biochem Sci.

[36] Pasparakis M (2012). Role of NF-κB in epithelial biology. Immunol Rev.

[37] Ghosh S, May MJ, Kopp EB (1998). NF-κB and rel proteins: evolutionarily conserved mediators of immune responses. Annu Rev Immunol.

[38] Zhu L-Y, Nie L, Zhu G, Xiang L-X, Shao J-Z (2013). Advances in research of fish immune-relevant genes: a comparative overview of innate and adaptive immunity in teleosts. Dev Comp Immunol.

[39] Netea MG, Simon A, van de Veerdonk F, Kullberg B-J, Van der Meer JW, Joosten LA (2010). IL-1β processing in host defense: beyond the inflammasomes. PLOS Pathog.

[40] Dinarello CA (2011). A clinical perspective of IL-1β as the gatekeeper of inflammation. Eur J Immunol.

[41] Jo A, Denduluri S, Zhang B, Wang Z, Yin L, Yan Z (2014). The versatile functions of Sox9 in development, stem cells, and human diseases. Genes Dis.

[42] Sarkar A, Hochedlinger K (2013). The sox family of transcription factors: versatile regulators of stem and progenitor cell fate. Cell Stem Cell.

[43] Agius C, Roberts RJ (2003). Melano-macrophage centres and their role in fish pathology. J Fish Dis.

[44] Steinel NC, Bolnick DI (2017). Melanomacrophage centers as a histological indicator of immune function in fish and other poikilotherms. Front Immunol.

[45] Manrique W, da Silva Claudiano G, Petrillo T, De Castro MP, Pereira Figueiredo M, de Andrade Belo M (2014). Response of splenic melanomacrophage centers of *Oreochromis niloticus* (Linnaeus, 1758) to inflammatory stimuli by BCG and foreign bodies. J Appl Ichthyol.

[46] Beso AJG, Candelaria VY, Cruz JGD, Tolentino MS, Tameta ADC, Espinosa AA. Effects of unleaded petroleum on the macrophage aggregates (MA) formation in red king tilapia (*Oreochromis* sp.) fingerlings. bioRxiv. 2016:044537. 10.1101/044537.

[47] Tjahjaningsih W, Pursetyo KT, Sulmartiwi L (2017). Melanomacrophage centers in kidney, spleen and liver: A toxic response in carp fish (*Cyprinus carpio*) exposed to mercury chloride. AIP Conf Proc..

[48] Manrique WG, Figueiredo MAP, Charlie-Silva I, de Andrade Belo MA, Dib CC (2019). Spleen melanomacrophage centers response of Nile tilapia during *Aeromanas hydrophila* and *Mycobacterium marinum* infections. Fish Shellfish Immunol.

[49] Bols NC, Brubacher JL, Ganassin RC, Lee LE (2001). Ecotoxicology and innate immunity in fish. Dev Comp Immunol.

[50] Popescu LM, Faussone-Pellegrini MS (2010). TELOCYTES - a case of serendipity: the winding way from interstitial cells of Cajal (ICC), via interstitial Cajal-Like cells (ICLC) to TELOCYTES. J Cell Mol Med.

[51] Mokhtar DM, Hussein MM (2020). Microanalysis of fish ovarian follicular atresia: a possible synergic action of somatic and immune cells. Microsc Microanal.

[52] Cretoiu SM, Popescu LM (2014). Telocytes revisited. Biomol Concepts.

[53] Huang YL, Zhang FL, Tang XL, Yang XJ (2021). Telocytes enhances M1 differentiation and phagocytosis while inhibits mitochondria-mediated apoptosis via activation of NF-κB in macrophages. Cell Transpl.

[54] Abdel-Maksoud FM, Hussein MM, Hamdy A, Ibrahim IA (2020). Anatomical, histological, and electron microscopic structures of syrinx in male budgerigars (*Melopsittacus undulatus*). Microsc. Microanal.

[55] Hussein MM, Mokhtar DM (2018). The roles of telocytes in lung development and angiogenesis: an immunohistochemical, ultrastructural, scanning electron microscopy and morphometrical study. Dev Biol.

[56] Wang M, Zhu Z (2019). Nrf2 is involved in osmoregulation, antioxidation and immunopotentiation in *Coilia nasus* under salinity stress. Biotechnol Biotechnol Equip.

[57] Ma Q (2013). Role of nrf2 in oxidative stress and toxicity. Annu Rev Pharmacol.

